# Effects of different prosthetic instrumentations on tibial bone resection in total knee arthroplasty

**DOI:** 10.1038/s41598-021-86787-x

**Published:** 2021-03-31

**Authors:** Yufeng Lu, Xuechao Yuan, Feng Qiao, Yangquan Hao

**Affiliations:** 1 Department of Integrated Traditional Chinese Medicine (TCM) and Western Medicine Orthopedics, Honghui Hospital, Xi’an Jiaotong University, Xi’an, 710054 Shaanxi People’s Republic of China; 2 Osteonecrosis and Joint Reconstruction Ward, Department of Joint Surgery, Honghui Hospital, Xi’an Jiaotong University, Xi’an, 710054 Shaanxi People’s Republic of China; 3 Shaanxi University of Chinese Medicine, Xianyang, 712046 Shaanxi People’s Republic of China

**Keywords:** Medical imaging, Prognosis, Therapeutics

## Abstract

Our aim was to assess the accuracy of the obtained posterior tibial slope (PTS) with a fixed angle cutting block. 247 TKAs in 213 patients were reviewed. We included 104 Legion Prosthesis, 76 U2 Knee Prosthesis, 46 NexGen LPS-Flex Prosthesis, and 21 Vanguard Knee System products. Preoperative and postoperative PTS were measured via expanded lateral tibia radiographs. For postoperative PTS, the Legion group had significantly smaller slopes than the U2 Knee group and Vanguard group. However, there was no significant difference between the Legion and NexGen groups, and no significant difference among the NexGen, U2 Knee, and Vanguard groups. Multiple linear regression showed that the different tibial lengths and preoperative PTS had statistically significant effects on postoperative PTS. However, there were weak correlations between the tibial length and PTS, and between preoperative and postoperative PTS. For TKA, although the PTS is not completely consistent with the angle of the cutting block, using conventional tibial bone resection technology with different tibial cutting instrumentations provided by various manufacturers in TKA can obtain safe PTS.

## Introduction

In total knee arthroplasty (TKA), the alignment of the sagittal plane of the prosthesis is as important as that of the coronal plane and axial position. Poor alignment can lead to early failure of the prosthesis^1,2^. The posterior tibial slope (PTS) relates to the postoperative range of motion^3^ and function of the extensor mechanism^4^. PTS also impacts tibial insert wear^5^ and loosening^6^, as well as the stability of the TKA^7^. Previous studies^8–10^ have suggested that the postoperative PTS should range from 0° to 10° to guarantee optimal prosthesis function. However, some authors^11–13^ recently recommended the reconstruction of the native PTS, depending on the intraoperative mobility and stability of the knee joint.

For TKA tibial bone resection, various manufacturers provide cutting blocks with certain PTS. In our institution, the commonly used TKA instrumentations are provided by Smith & Nephew, United Orthopedic, Zimmer, and Biomet with angles of 3°, 5°, 7°, and 0° respectively.

In the current study, we aimed to assess the PTS after different tibial cutting instrumentations were employed for TKA. We hypothesized that the PTS achieved after osteotomy with different extramedullary guidance jigs are inconsistent with the fixed angle of the cutting blocks.

## Patients and methods

The protocols described herein were approved by the ethics reviewing council of Honghui Hospital, Xi'an Jiaotong University, which abides by the Declaration of Helsinki on Ethical principles for medical research involving human subjects (IRB Approval Number 202003058). Written informed consent was obtained from all participants.

This study retrospectively reviewed 320 TKAs performed by the senior surgeon (HY) using posterior-stabilizing prostheses between January 1, 2018, and December 31, 2019. The inclusion criteria were as follows: (1) preoperative diagnosis of knee osteoarthritis; and (2) true knee lateral radiographs, including at least 20 cm of the tibia. Exclusion criteria included (1) evidence of trauma, infection, tumor, or any congenital disorder; and (2) tibial plateau with severe bone defect(s).

Using these criteria, 247 knees of 213 patients (155 women and 58 men) were included. There were 122 left knees and 125 right knees. The mean patient age at the time of index operation was 62.5 years (range, 30–87 years). The mean follow-up was 15.3 months (range, 6–24 months). There were 104 Legion Prostheses (Smith & Nephew, Memphis, TN), 76 U2 Knee Prostheses (United Orthopedic, Taipei, Taiwan), 46 NexGen LPS-Flex Prostheses (Zimmer, Warsaw, IN), and 21 Vanguard Knee Systems (Biomet, Warsaw, IN) used.

### Surgical technique

**Figure 1:**
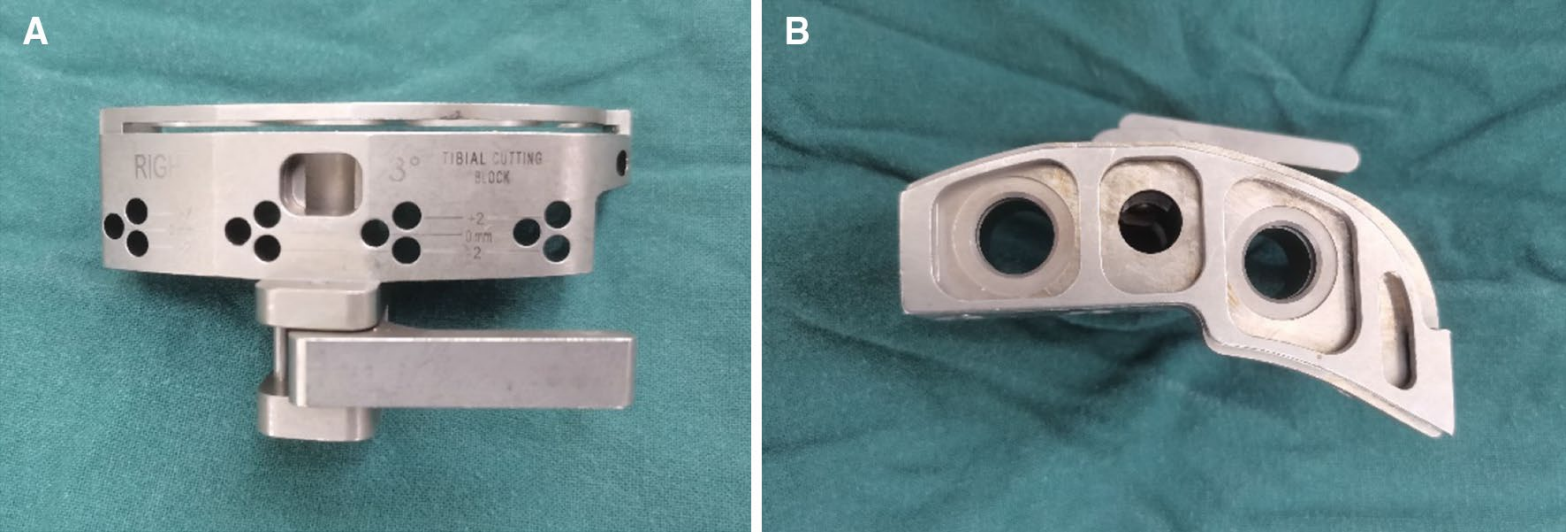
** (A,B**) Tibial cutting block provided by Smith & Nephew has an angle of posterior inclination of 3° for the Legion system.

**Figure 2:**
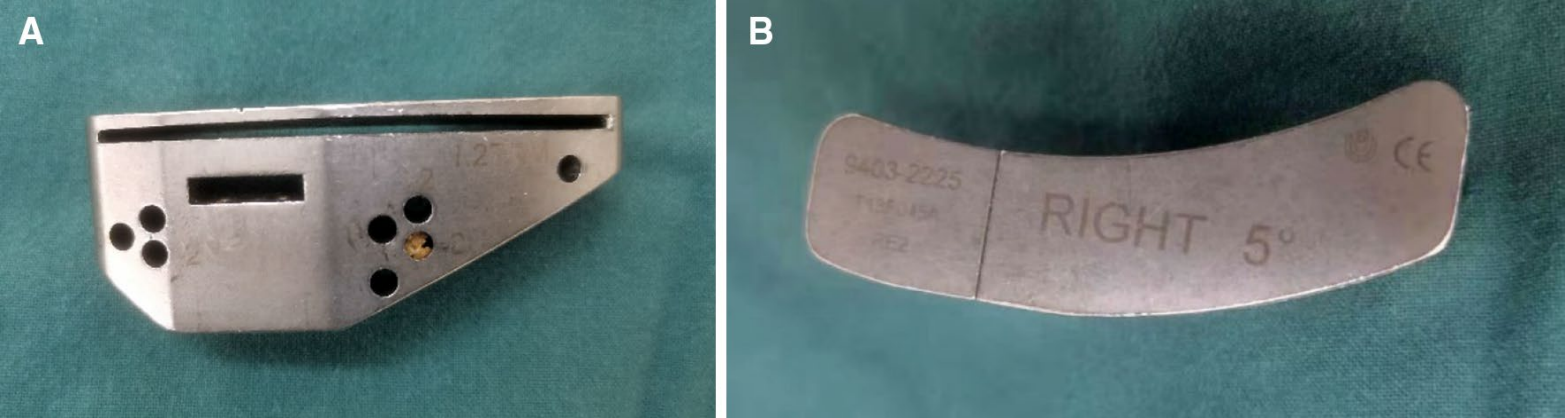
**(A,B)** Tibial cutting block provided by United Orthopedic has an angle of posterior inclination of 5° for the U2 Knee system.

**Figure 3:**
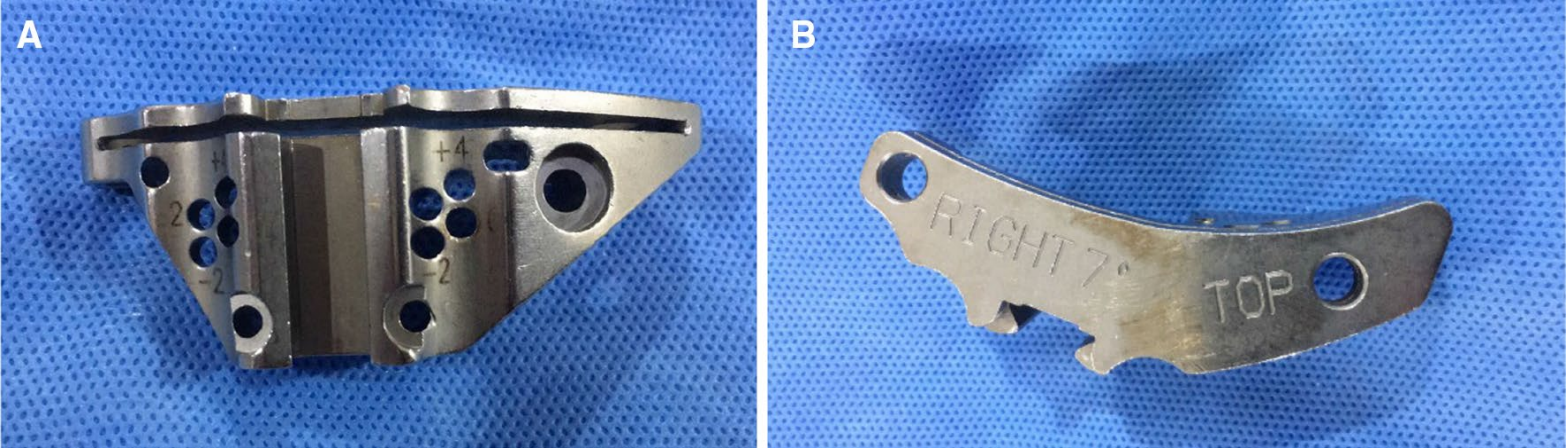
**(A,B)** Tibial cutting block provided by Zimmer has an angle of posterior inclination of 7° for the NexGen system.

**Figure 4:**
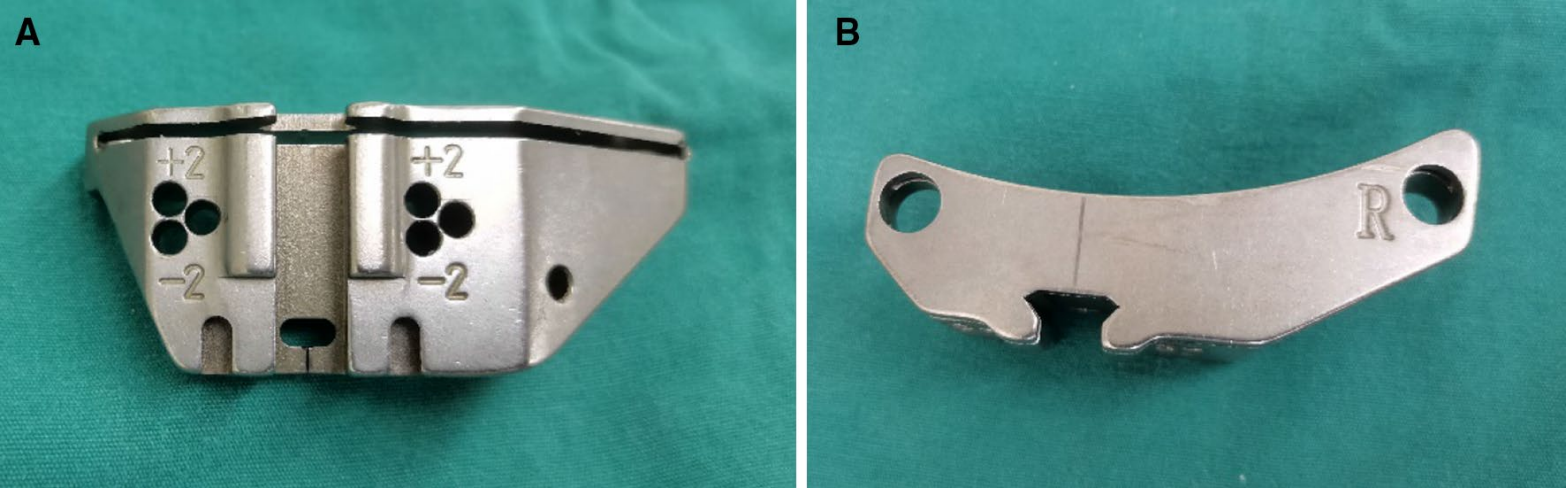
**(A,B)** Tibial cutting block provided by Biomet had an angle of posterior inclination 0° for the Vanguard system.

All TKAs were performed by the senior surgeon (HY) using a midline skin incision and medial parapatellar arthrotomy. The prosthesis was chosen by the patient according to their own situation, and none of the PTS of patients were measured before surgery. The tibia cuts were made with the use of an extramedullary guidance jig. After resection of the distal femur, the tibia was anteriorly subluxated, and the tibial alignment guide and cutting block were assembled. The guide spike was anchored to the ACL attachment of the tibia. The cutting jig was fixed to the proximal tibia by two parallel pins and one oblique pin. In the coronal plane, the alignment guide pointed to the second metatarsal, and the proximal tibia was cut perpendicular to the guide in the coronal plane. In the sagittal plane, the surgeon used his fingers to determine the PTS osteotomy. At the lower edge of the tibial tuberosity, the anterior side of the tibia was two fingers’ width from the guide, and, at the upper side of the ankle joint clamp, the anterior tibial skin was three fingers’ width from the guide rod. Each tibial cutting block provided by the manufacturers had an angle of posterior inclination, which was 3° for the Legion system (Fig. 1A,B), 5° for the U2 Knee system (Fig. 2A,B), 7° for the NexGen system (Fig. 3A,B), and 0° for the Vanguard system (Fig. 4,B).

**Figure 5:**
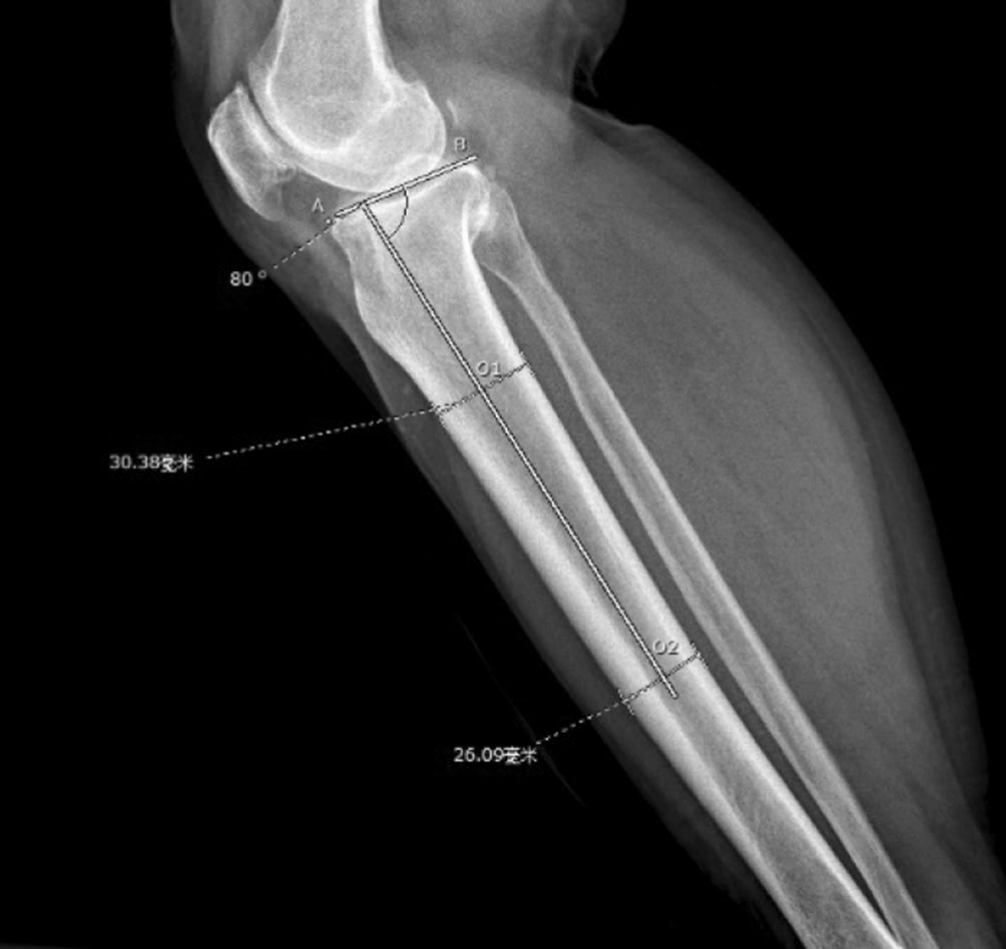
A is the anterior edge of the tibial plateau, B is the posterior edge of the tibial plateau, O1 and O2 are the midpoints of the anterior and posterior cortical edges 6 cm and 16 cm distal from the tibial plateau respectively. The preoperative PTS is 90° minus the angle between the line AB and the line O1O2.

**Figure 6:**
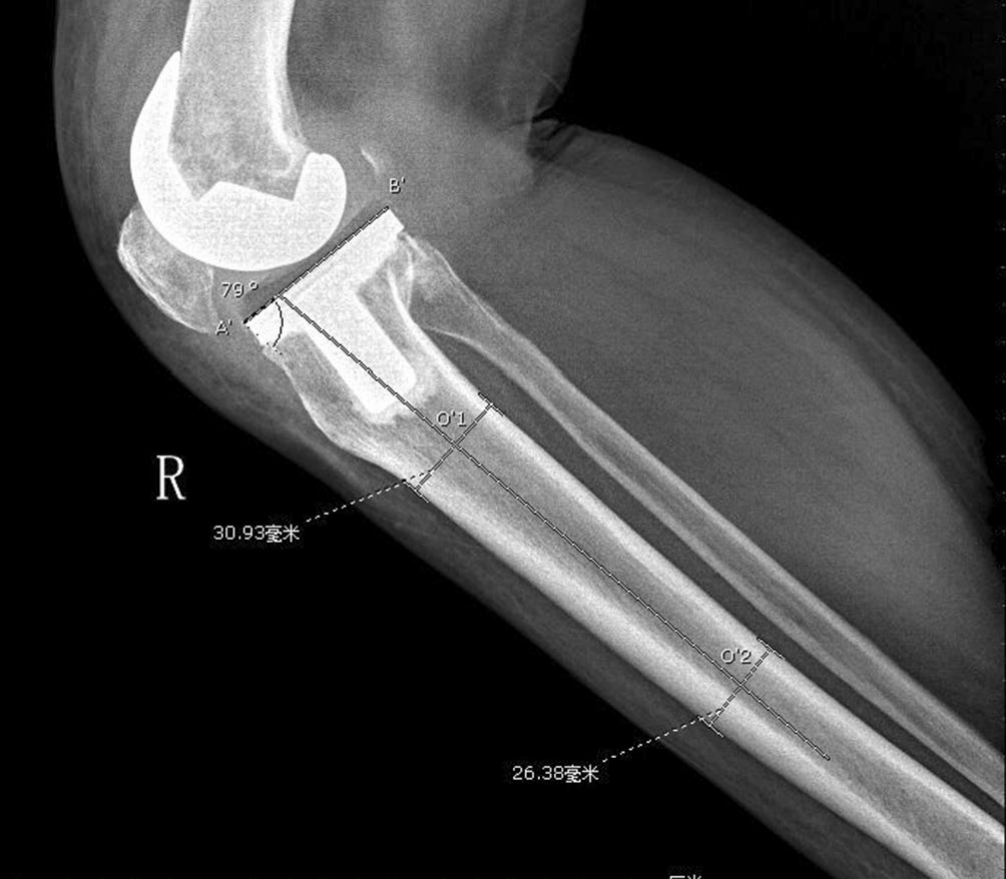
A’ is the anterior edge of the tibial component, B’ is the posterior edge of the tibial component, O’1and O’2 are the midpoints of the anterior and posterior cortical edges 6 cm and 16 cm distal from the tibial component respectively. The postoperative PTS is 90° minus the angle between the line A’B’ and the line O’1O’2.

**Figure 7:**
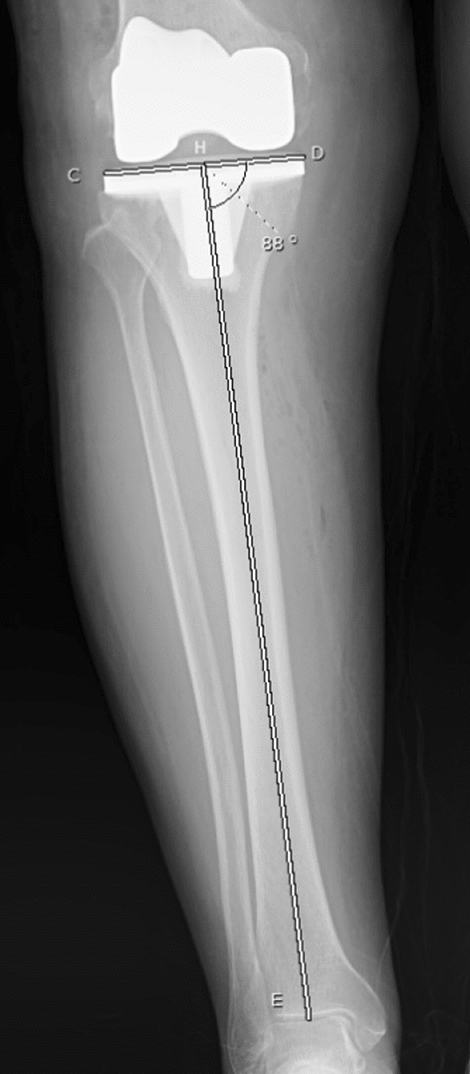
C is the lateral edge of the tibial component, D is the medial edge of the tibial component, H is the midpoint of the CD, E is the center of the ankle joint, and the angle between CD and HE is the tibial component coronal alignment.

**Figure 8:**
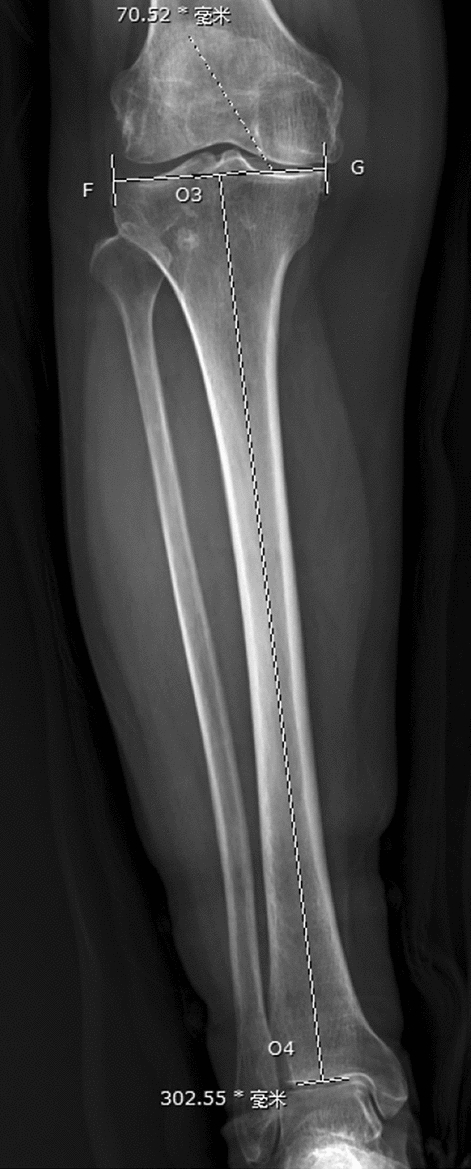
F is the lateral edge of the tibial plateau, G is the medial edge of the tibial plateau, O3 is the midpoint of FG, O4 is the midpoint of the distal tibial articular surface, and the length of O3O4 is the tibia length.

All measurements were carried out with a picture archiving and communication system (PACS, Synapse, Fujifilm Inc., Tokyo, Japan). (1) PTS was measured according to Faschingbauer's method^14^ using the true knee lateral radiographs. The anatomic axis of the tibia was taken as the line connecting the midline of the anterior and posterior (AP) cortical edges 6 cm and 16 cm distal from the tibial plateau. The AP axis of the tibial plateau was the line connecting the AP edges of the tibial plateau. If there was an obvious osteophyte on the AP edge of the tibial plateau, the medial plateau was used as the AP axis. The preoperative PTS was 90° minus the angle between the two axes (Fig. 5). The postoperative PTS was 90° minus the angle between the anatomic axis of the tibia and the AP axis of the tibial component (Fig. 6). (2) The tibial component coronal alignment angle (TCCA) was defined as the angle between the mechanical axis of the tibia and transverse axis of the tibial component on postoperative standing full-length AP radiographs (Fig. 7). (3) The tibia length was defined as the distance between the midpoint of the proximal tibial articular surface and the midpoint of the distal tibial articular surface on preoperative standing full-length radiographs (Fig. 8).

The measurement data were divided into four groups according to prosthesis type: Legion group, U2 Knee group, NexGen group, and Vanguard group. All measurements were performed by two blinded observers (LY and YX) using radiographs. After 3 weeks, 20 randomly selected patients were measured again for the determination of intra-rater and inter-rater reliability.

Clinical outcome assessment used the knee social score (KSS) pre-operatively and at final follow-up. The KSS comprises two parts: a knee score, which includes pain, stability, and range of motion (ROM) and a function score, which includes the patient’s ability to walk and climb stairs, and the need for ambulatory aids.

Quantitative data were expressed as means ± standard deviation (SD). Statistical analyses were performed using the PASW statistics 18 (SPSS Inc., Chicago, IL, USA).

The normality assumption of our data was validated by the Kolmogorov–Smirnov test. A one-way ANOVA test and Kruskal–Wallis non-parametric tests were used to compare the data for the four groups. Intra- and inter-rater reliability^15^ were determined using the intraclass correlation coefficient (ICC). Multiple linear regression analysis was used to investigate the possibility of association of age, sex, body side, TCCA, tibial length, preoperative PTS, preoperative and postoperative KSS as well as postoperative ROM with postoperative PTS. P < 0.05 was considered to be statistically significant.

## Results

The Kolmogorov–Smirnov test revealed that all data followed a normal distribution pattern. ICC and interclass correlation coefficients for the reproducibility of all parameters were > 80% (Table 1).

**Table 1:** The intraclass correlation coefficient analysis of the measured data.

Measurement	Intra-observer I reliability	Intra-observer II reliability	Inter-observer reliability
Post-op. PTS	0.93	0.90	0.91
Pre-op. PTS	0.88	0.90	0.82
TCCA (°)	0.87	0.94	0.89
Tibia length	0.99	0.99	0.99

*PTS* posterior tibial slope, *TCCA* tibial component coronal alignment angle.

There were no statistically significant differences among the groups with regards to the demographic characteristics of patients before surgery, except that the age of the U2 Knee group was greater than the other groups (Table 2). There were also no statistically significant differences among the four groups with regards to preoperative and postoperative KSS and ROM (Table 2). The homogeneity of variance test indicated that there was no statistically significant difference among the corresponding data of each group (all P-values > 0.05) (Table 3).

**Table 2:** Summary of patient and clinical results.

	Legion	U2 knee	NexGen	Vanguard	
Age(years)	66.0 ± 7.8	70.9 ± 6.1	64.7 ± 7.3	63.7 ± 7.7	< 0.001*
Gender (m/f)	24/65	19/49	11/29	4/12	0.996
Side (l/f)	45/59	39/37	24/22	14/7	0.230
Height (cm)	157.9 ± 10.5	157.2 ± 9.4	159.5 ± 9.2	158.3 ± 9.0	0.670
Weight (kg)	64.1 ± 11.1	62.9 ± 9.7	65.7 ± 10.1	63.6 ± 9.4	0.555
BMI (kg/m^2^)	25.5 ± 2.3	25.3 ± 2.5	25.2 ± 4.1	25.2 ± 2.1	0.962
**Pre-op. KSS**					
Knee score	37.4 ± 11.4	35.5 ± 11.9	38.1 ± 11.1	37.1 ± 12.0	0.619
Function score	41.3 ± 9.4	39.1 ± 10.3	40.1 ± 11.2	37.3 ± 12.4	0.300
**Post-op. KSS**					
Knee score	90.2 ± 4.1	90.1 ± 5.4	89.9 ± 5.3	90.8 ± 5.4	0.899
Function score	85.0 ± 8.3	85.1 ± 8.4	87.3 ± 8.8	85.2 ± 9.8	0.456
Pre-op. ROM	74.2 ± 18.2	75.3 ± 15.9	73.4 ± 16.4	74.2 ± 16.6	0.938
Post-op. ROM	112.4 ± 12.5	111.9 ± 12.2	113.1 ± 15.5	111.5 ± 14.7	0.959

*BMI* body mass index, *KSS* Knee Society score, *ROM* range of motion.

*U2 Knee was significantly different from those of other groups.

**Table 3:** Measurement data and homogeneity of variance test of four kinds of prosthesis instrumentation.

Group	n	Pre-op. PTS (°)	Post-op. PTS (°)	TCCA (°)	Tibia length (cm)
Legion	104	11.6 ± 4.7	6.3 ± 2.6	89.5 ± 1.8	34.2 ± 2.5
U2 knee	76	10.8 ± 4.9	7.6 ± 2.6	89.5 ± 1.9	34.0 ± 2.6
NexGen	46	10.0 ± 5.8	7.1 ± 2.7	89.6 ± 1.8	35.0 ± 2.3
Vanguard	21	11.0 ± 4.4	8.6 ± 3.3	88.5 ± 2.6	34.8 ± 2.6
P		0.628	0.768	0.088	0.742

*PTS* posterior tibial slope, *TCCA* tibial component coronal alignment angle.

**Figure 9:**
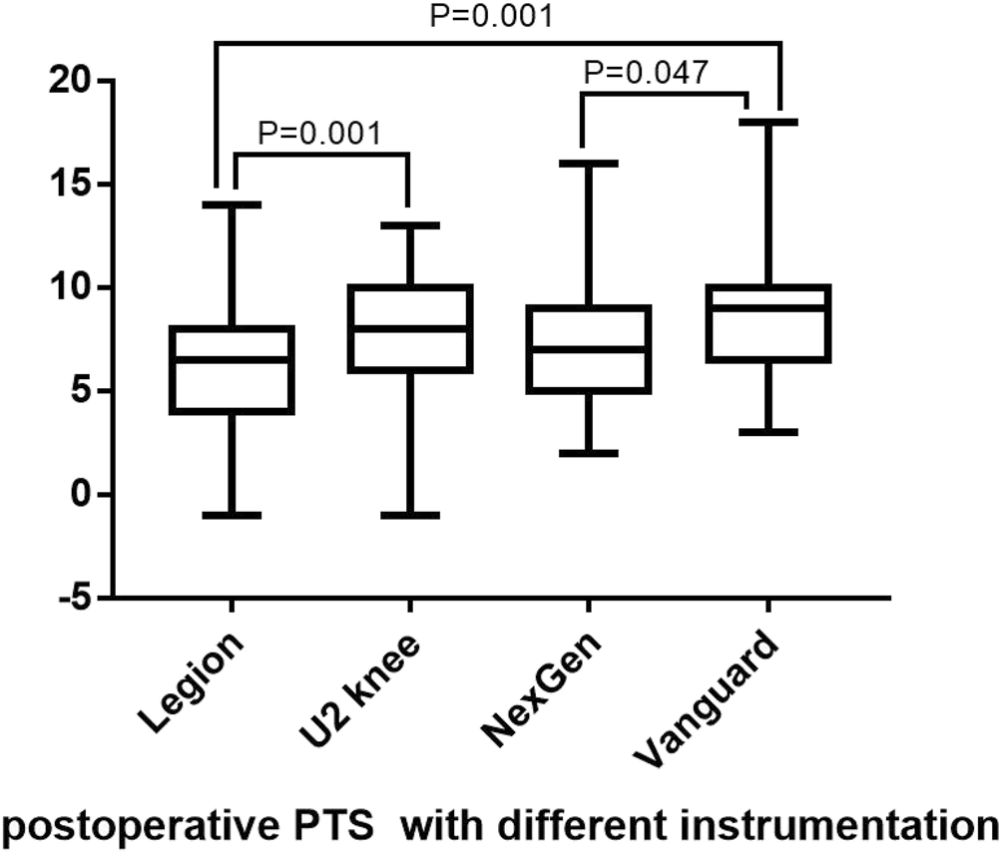
A boxplot illustrating the distributions of the four postoperative PTS.

For the preoperative PTS and the tibia length, there were no significant differences among the four groups (all P-values > 0.05). For postoperative PTS, the Legion group had a significantly smaller slope than the U2 Knee and Vanguard groups (P = 0.001) (Fig. 9). However, there was no significant difference in postoperative PTS between the Legion and NexGen groups (P = 0.08), and no significant differences were found among the NexGen, U2 Knee, and Vanguard groups (all P-values > 0.05).

There was no significant difference in TCCA among the four groups (all P-values > 0.05). However, a one sample t-test was performed for the TCCAs of each group and 90°, and only the NexGen group showed no significant difference from 90° (t = -1.421, P = 0.162). Furthermore, the other prosthetic instruments and their PTS showed significant differences (all P < 0.05) (Table 4).

**Table 4:** One-sample t-test results for the postoperative PTS compared with the fixed angle provided by the cutting block plus 3° and between the TCCAs and 90°.

Group	Post-op. PTS (°)	Fixed angle + 3 (°)	t	P	TCCA (°)	90°	t	P
Legion	6.3 ± 2.6	6	1.199	0.233*	89.5 ± 1.8	90	−2.337	0.021
U2 Knee	7.6 ± 2.6	8	−0.998	0.321*	89.5 ± 1.9	90	−2.213	0.030
NexGen	7.1 ± 2.7	10	−6.967	< 0.001	89.6 ± 1.8	90	−1.421	0.162*
Vanguard	8.6 ± 3.3	3	7.644	< 0.001	88.5 ± 2.6	90	−2.464	0.023

*PTS* posterior tibial slope, *TCCA* tibial component coronal alignment angle.

*P > 0.05.

Using the conventional tibial bone resection technology of two fingers proximally and three fingers distally. Empirically, it added about 3 degrees of posterior tilt. Therefore, A one sample t-test was performed for the postoperative PTS of each group and the fixed angle provided by the prosthetic instrumentations plus 3°, and the results indicated that only the Legion group (t = 1.199, P = 0.233) and the U2 Knee group (t = -0.998, P = 0.321) showed no statistical difference from the built-in posterior tilt angle provided by the product, whereas the PTS of the other prosthetic instrumentations were significantly different their specific fixed angles (all P < 0.05) (Table 4).

Multiple linear regression showed that the tibial length and preoperative PTS had statistically significant effect on postoperative PTS (b =  − 0.023 and 0.093, t =  − 3.474 and 2.679, P = 0.001 and 0.008, respectively) (Table 5); although, there were weak correlations between the tibial length and postoperative PTS (R = 0.255, P < 0.001), and between preoperative PTS and postoperative PTS (R = 0.210, P = 0.001). The different ages, TCCAs, BMI, postoperative KSS, and ROM had no significant effect on PTS (Table 5).

**Table 5:** Multiple linear regression of influencing factors on postoperative PTS.

Independent variable	Unstandardized coefficient b	Standard error	Standardized coefficient b	t value	P Value
Constant	19.466	9.095	–	2.140	0.033
Pre-op. PTS	0.093	0.035	0.165	2.679	0.008*
TCCA	−0.129	0.087	−0.089	−1.484	0.139
Tibia length	−0.023	0.007	−0.212	−3.474	0.001*
BMI	−0.076	0.062	−0.074	−1.230	0.220
Post-op. KSS (knee score)	0.046	0.070	0.080	0.664	0.507
Post-op. KSS (function score)	−0.008	0.037	−0.023	−0.204	0.839
Post-op. ROM	0.041	0.022	0.191	1.848	0.066

Dependent variable: post-op. PTS.

*PTS* posterior tibial slope, *TCCA* tibial component coronal alignment angle, *BMI* body mass index, *KSS* Knee Society score, *ROM* range of motion.

*p < 0.05.

## Discussion

The PTS is defined as the angle between the tangent of the medial and lateral plateaus and the line perpendicular to the longitudinal mechanical axis^14^. This angle is very different between individuals and ranges in studies from –9° to 16° with an average of approx. 3°–10°^16–18^.

Currently, the most commonly used method for measuring the PTS is the true lateral radiograph of the tibia. For optimal determination, a strictly lateral radiograph of the entire length of the tibia, including the ankle and knee joint, is required^14^. The mechanical axis (TLA) is constructed from a line between the center of the tibial plateau and that of the lateral ankle joint. A tangent is placed over the tibial plateau, and the angle between the mechanical axis and the tangent determines the PTS. However, in clinical practice, before and after TKA, full-length lateral radiographs of the tibia are not routinely available, so there are many alternatives for the tibial mechanical axis, such as the tibial proximal anatomical axis^18–22^, tibial shaft anatomical axis^18,23^, posterior tibial cortex^18,20,22^, anterior tibial cortex^18–20,22^, and fibular shaft axis^18,23^. Compared with the tibial mechanical axis, the accuracy of the PTS measurements varied. Current studies show there is good correlation between the tibial proximal anatomical axis, constructed by measuring points 5–15 cm or 6–16 cm below the joint surface, and the mechanical axis. In this way, deviations can be reduced to up to 1.5°^14,18^. The shorter the radiograph that includes the tibia, the worse the PTS measurement accuracy tends to be in these studies. Therefore, based on the results of these studies, we used an expanded lateral radiograph of a at least 20-cm long section of the tibia to measure PTS in this study. Obviously, the accuracy of this measurement is lower than that obtained when using the full-length lateral tibia, which is a limitation of this study. However, according to the literature^14,18^, this was the alternative method with the smallest error.

Appropriate PTS for TKA is very important. Previous studies suggested that postoperative PTS should range from 0° to 10° to guarantee optimal prosthetic function. Excessive PTS after TKA may cause anterior and posterior instability, leading to anterior subluxation of the tibia, thus increasing the shear stress of the posterior tibia polyethylene and resulting in aseptic loosening^22^. Conversely, a reduction in the PTS leads to increased stress in the anterior part of the subchondral bone, thereby increasing the risk of component subsidence^24^. Decreased PTS also leads to limited flexion because of the tight flexion gap^25^.

The method used for tibial bone resection primarily depends on the implant instrumentation provided by the manufacturer. In the coronal plane, the tibial bone resection needs to be perpendicular to the tibial mechanical axis. In addition to navigation and patient-specific instrumentation, the traditional method of aligning the tibial mechanical axis is to use the proximal spike of the cutting guide to anchor the ACL attachment to the tibia^26^, the anterior middle third of the anterior and posterior axis of the tibial plateau^27^, and the intercondylar eminentia^28^, resulting in the extramedullary rod being parallel to the palpable fibula^27^. In the distal tibia, because the ankle joint center is difficult to locate, the second metatarsal^29^, first and second metatarsal spaces^30^, tibialis anterior tendon, or anterior tibial crest^31,32^ are often used as markers, and through these, generally good coronal alignment can be obtained. The sagittal mechanical axis of the tibia is more difficult to mark than the coronal mechanical axis. Therefore, the specific method of bone resection of the PTS is still controversial, and there is no unified standard. The traditional method is to adjust the PTS using the distance between the tibial cutting guide rod and the anterior skin surface of the tibia as a reference^33^. The accuracy of the cutting block with a fixed angle posterior slope provided by the manufacturer was uncertain. Therefore, the present study used conventional tibial bone resection techniques to compare the actual PTS obtained by various makes of cutting blocks with fixed angles to test their accuracy. We found that when we used a 3° cutting block (Legion), the angle after bone resection was 6.3° ± 2.6°. For a 5° cutting block (U2 Knee), the angle after osteotomy was 7.6° ± 2.6°. With the 7° cutting block, the angle after osteotomy was 7.1° ± 2.7° (NexGen). Surprisingly, for the Vanguard’s 0° cutting block, the angle after resection was 8.6° ± 3.3°. We performed multiple linear regression analysis of the PTS with the parameters of age, TCCA, BMI, preoperative PTS, postoperative KSS, ROM, and tibial length, and found that only the tibial length and preoperative PTS affected the PTS, although, the effect was very small (R = 0.255 and 0.210, respectively). However, considering that we used the expanded lateral radiograph with a 20-cm section of the tibia instead of the full-length lateral tibial radiograph, the actual PTS may be 1°–1.5° less than the above value. In addition, in the coronal plane, we used the second metatarsal bone to align the cutting guide rod, and the TCCAs obtained with these four prosthetic instrumentations were 89.5°–89.6°; only the NexGen group had no statistical difference from 90°, while the other three groups all showed statistical difference from 90°. This suggests that the second metatarsal bone is not a reliable reference marker for coronal tibial bone resection with some instrumentations, as it is more easily affected by the position of the ankle joint.

There were several limitations to our study. First, different surgeons have different finger widths, and using this traditional surgical measurement technique may cause the distance between tibial cutting guide rod and the anterior edge of the tibia to be different between different surgeons, resulting in different PTS. Although all operations in this study were performed by the same surgeon, which minimized the bias caused by the surgical technique, the study did not consider the influence of the finger widths of different surgeons on the tibial osteotomy. Second, because of the use of the extended tibial lateral radiograph instead of full-length lateral radiograph of the tibia, the PTS obtained was reduced by an average of 1.5 degrees. Even so, the posterior tibia obtained by using Legion, U2 Knee, and Vanguard instrumentations were all greater than the angles of the fixed angles on the cutting block, which means that the PTS obtained using these four instrumentations were greater than those using the cutting block. However, the angle after resection for the Vanguard’s 0° cutting block was 8.6° ± 3.3° and thus had a significantly greater margin of error than the other three instrumentations. The reasons may be that (1) the sample size was too small, thus a larger sample is needed for future verification, and (2) the anatomical features of the proximal tibia may differ between East Asians and Caucasians, and knee deformity is usually more pronounced in Asian than Caucasian patients at the time of TKA. Nevertheless, according to the postoperative KSS and knee ROM, most of the patients obtained satisfactory results. Therefore, although the PTS is not completely consistent with the angle of the cutting block, using conventional tibial bone resection technology with different tibial cutting instrumentations provided by various manufacturers in TKA can obtain safe PTS.
